# Perioperative Nerve Blockade: Clues from the Bench

**DOI:** 10.1155/2011/124898

**Published:** 2011-07-12

**Authors:** M. R. Suter, A. Siegenthaler, I. Decosterd, R. R. Ji

**Affiliations:** ^1^Pain Center, Department of Anesthesiology, University Hospital Center and University of Lausanne, 1011 Lausanne, Switzerland; ^2^Department of Cell Biology and Morphology, University of Lausanne, 1011 Lausanne, Switzerland; ^3^Pain Research Center and Department of Anesthesiology, Brigham and Women's Hospital and Harvard Medical School, Boston, MA 02115, USA

## Abstract

Peripheral and neuraxial nerve blockades are widely used in the perioperative period. Their values to diminish acute postoperative pain are established but other important outcomes such as chronic postoperative pain, or newly, cancer recurrence, or infections could also be influenced. The long-term effects of perioperative nerve blockade are still controversial. We will review current knowledge of the effects of blocking peripheral electrical activity in different animal models of pain. We will first go over the mechanisms of pain development and evaluate which types of fibers are activated after an injury. In the light of experimental results, we will propose some hypotheses explaining the mitigated results obtained in clinical studies on chronic postoperative pain. Finally, we will discuss three major disadvantages of the current blockade: the absence of blockade of myelinated fibers, the inappropriate duration of blockade, and the existence of activity-independent mechanisms.

## 1. Introduction

The major interest of regional anesthesia (central or peripheral nerve block) in clinical practice remains to replace or supplement general anesthesia for certain types of surgery and to provide efficient pain relief in the postoperative period. Being able to continue the block in the postoperative period allows a more effective pain control and a reduction of opioid-related side effects [1, 2]. While the improved patient comfort is undoubted, an overall improvement in long-term patient outcome is less evident and combining general anesthesia with a regional analgesic technique exposes the patient to the risk of both techniques. Since the concept of pain treatment as a fundamental human right has emerged, the use of invasive pain treatment is warranted already for the improvement of acute postoperative pain treatment [3]. Efficacy of nerve blockade on serious complications in the postoperative period (major morbidity or mortality) is hard to demonstrate because of their low incidence [4]. The possibility of preventing chronic postsurgical pain is becoming a major issue [3, 5, 6]. In the clinical research literature, there are only few reports showing a benefit of regional analgesic techniques on the incidence of chronic postoperative pain states. Two RCT demonstrated a reduction of the incidence of chronic postthoracotomy pain in patients treated with peri- and postoperative epidural analgesia as compared to patients without postoperative epidural analgesia [7, 8]. Further, a single paravertebral block reduced the incidence of postmastectomy pain one year following surgery as compared to a sham puncture [9]. Whereas the incidence of phantom limb pain after amputation was not influenced by epidural analgesia [10]. Surrogate outcomes are studied to increase the indications of regional analgesic techniques: length of hospital stay, improvement on long-term function after surgery (in orthopedics) and, newly, cancer recurrence [11], and reduction in surgical site infection [12]. Experimental research on humans and animals could help to define more clearly the working hypothesis of clinical trials and their design.

Current pain treatment should use a mechanistic-based approach. The best mechanistic knowledge in patient to-date is obtained from quantitative sensory testing (QST) where different modalities of sensation are tested. There is a gap between the sensory testing in human and the cellular and molecular vision offered in an experimental laboratory setting. The experimental setting also allows different types of blockade, using drugs with specificities on nerve fiber types, longer duration, or motor blockade, all of which cannot be tested on patients unless strong evidence has been gathered beforehand in experimental studies. 

This paper is therefore intended to focus on the effect of nerve blockade on pain-related behavior and central changes that occur after peripheral tissue injury in animals (e.g., rats and mice) and build a bridge to clinical practice. We will try to point out some discoveries in bench research that would answer questions or lead to research ideas around the operating room.

## 2. Pathophysiology of Peripheral Discharges and Central Mechanisms of Persistent Pain

### 2.1. Peripheral Activity and Central Sensitization: A Potential Contribution to Chronic Postoperative Pain

The potential benefits of regional analgesic techniques rely on the ability of local anesthetics to reduce or abolish the peripheral input electrically transmitted by the nerve. Tissue injury and/or inflammation (with potential nerve lesion) during surgery lead to a massive input of action potentials along the primary afferents. The first relay of the information in the central nervous system (CNS) is the spinal cord dorsal horn. The glutamate release at the synapses in the dorsal horn induces a depolarization in the second-order neuron. If its amplitude is large enough, it triggers an action potential that conducts the information to higher centers. Cumulative afferent inputs gradually sensitize second-order neurons, which become more reactive to subsequent inputs. This global process of signal enhancement in the CNS is called central sensitization and encompasses increased membrane excitability, synaptic efficacy, and reduced inhibition. Central sensitization and its dependency on primary afferent activity has been extensively reviewed by Latremoliere and Woolf [13]. It is often described in 2 temporal phases an early short lasting, transcription-independent phase caused by phosphorylation mechanisms and a late longer lasting phase dependant on transcription and synthesis of new proteins [14]. Central sensitization is triggered by primary afferent release of glutamate which binds on postsynaptic ionotropic (AMPA (amino-3-hydroxy-5-methyl-4-isoxazole propionate), NMDA (N-methyl-D-Aspartate) and kainate), and metabotropic (mGluR 1–8, not all expressed in spinal cord) receptors. Under normal stimulation, the NMDA receptor is blocked by a magnesium (Mg^++^) ion in a voltage-dependent manner. Following sustained activity as in the case of surgery, the Mg^++^ block is released and glutamate can open the NMDA receptor leading to greater calcium entry in the spinal cord neuron, the first step of central sensitization. This is enhanced by the neuropeptides Substance P and CGRP, also released from primary afferents. The increase in cellular calcium in the dorsal horn neuron appears to be the trigger for the next step of central sensitization implicating activation of kinases (protein kinase A (PKA), C (PKC), or calmodulin kinase II (CaMKII)). These kinases phosphorylate different channels thereby increasing their trafficking to the membrane or changing their biophysical properties globally enhancing their response. Other targets in the later phases of the sensitization phenomenon include mitogen-activating kinases (MAPKs) such as extracellular signal-regulated kinase (ERK) and transcription factors finally leading to changes in gene expression.

### 2.2. Characterization of Spontaneous Discharges after Nerve Injury

**Figure 1:**
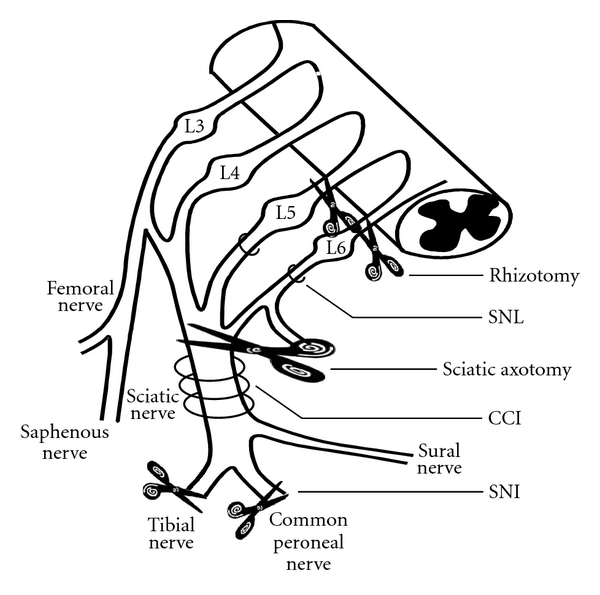
Schematic of the major animal models of nerve injury. Rhizotomy consists of section of dorsal roots; SNL: spinal nerve ligation, usually L5 or L5 and L6; CCI: chronic constriction injury consists of loose ligations of the sciatic nerve; SNI: spared nerve injury, consists of section of the tibial and the peroneal branches leaving the sural intact.

Spontaneous activity occurs from the neuroma (unregulated regeneration of the nerve stump after injury) after nerve section [15, 16], and there have been numerous descriptions of increased peripheral activity after nerve injury, in different neuropathic pain models (Figure 1) and at different timepoints after injury. Most agree that ectopic activity in primary afferent after nerve injury arises from multiple sites (the neuroma, along the nerve, or in the dorsal root ganglion (DRG)) [17, 18]. However, there are still controversies about which type of fibers (injured versus noninjured fibers or myelinated versus unmyelinated fibers) [17]. Since the early recordings following axotomy, different animal models of neuropathic pain (Figure 1) were developed, many consisting of partial nerve lesions. They lead to various configurations between intact and injured nerve fibers. In chronic constriction injuries (CCI, originally described by Bennett and Xie [19] and modified by Mosconi and Kruger [20]), mostly myelinated fibers are injured, leaving neighboring C-fibers relatively undamaged [21]. In the spinal nerve ligation model (SNL) model [22], intact roots are in contact distally with the degenerating fibers of the injured roots and in the spared nerve injury (SNI) model [23] intact fibers are in contact with the proximal part of the injured nerves. 

Summarizing all studies on peripheral nerve activity recordings is difficult but we will take out the general ideas. Most researchers consider A-fibers as the principal contributors to peripheral ectopic firing following nerve injury [24–28]. Nevertheless, activity in the unmyelinated C-fibers was recorded either very early, during the first 15 minutes after a nerve lesion [29], or later, after a few days [30]. C-fiber activity was also recorded after spinal nerve ligation in the neighbored intact spinal nerve [31] or after stimulation of a nerve stump with nociceptive mediators [32]. This underlines the importance of uninjured fibers as provider of afferent inputs or of aggravating factors that we could also use as potential therapeutic target [25, 33, 34]. 

Is pain-related behavior linked to this ectopic firing? Indeed in neuropathic models, onset of activity is strongly related to the generation of pain [17, 31, 35, 36]. Ectopic discharges were even correlated with pain-related behavior at the early phase of nerve injury but not later on [37].

In a translational perspective, a nerve blockade, peripheral or neuraxial, should therefore cover impulse from both myelinated and unmyelinated fibers in the postoperative period. The minimal timeframe until peripheral input is no longer associated with pain-related behavior after surgery still has to be defined. Interestingly, for the clinical setting, Brennan's group recently paralleled guarding behavior in rodents to spontaneous pain in postoperative patient. They were able to show that skin plus deep tissue incision induces a guarding behavior and increased spontaneous afferent activity which was not present in skin incision alone [38]. This brings to attention that spontaneous activity can appear in an inflammatory model without obvious nerve injury as seen in our daily surgical activity.

## 3. Advantages and Limits of Animal Research

The necessity of animal models has always been criticized [39, 40]. A few well-known failures to translate research findings into clinical trials, NK-1 antagonists [41], glycine site antagonist [42], or sodium channel blockers [43] remind us of the potential gaps between animals and humans. On the other hand, examples of successful translational research exist such as conotoxin, which revealed a new mechanism in pain development and lead to a new treatment [44]. New compounds are coming to clinical trial, nerve growth factor (NGF) inhibitor [45, 46], or transient receptor potential vanilloid receptor 1 (TRPV1) antagonists [47, 48].

Three current limitations in humans are cited by Mogil [40], (i) single neuron recording which gives valuable information is not obtainable in human, (ii) functional magnetic resonance imaging reaches a ceiling and high activity in neurons cannot be differentiated, and (iii) some regions of interest such as the spinal cord dorsal horn or the DRG are too small to be seen clearly. Therefore, most human studies characterize pain states and do not look at anatomical, biochemical, or physiological mechanisms. We are however able to see an increase in imaging studies which, together with quantitative sensory testing, represent the best mechanistic approach feasible in living patients and with technical improvement some limitations seen above will be overcome. 

An obvious advantage of animals is the standardization of the injury and of the genetic and environmental background and avoiding any social factors. Nerve injuries on patients are heterogeneous and, therefore, difficult to study. We will here highlight the advantages and the limits of animal research specifically looking at nerve blockade issues.

### 3.1. Advantages of Animal Research

**(a) Sustained Block**

The influence of duration and initiation of epidural nerve blockade to prevent chronic changes after amputation has been studied clinically already 20 years ago with controversial results [10, 49]. For local anesthetics, delivery through slow release polymer is complicated and therefore peripheral or central nerve blockade lasting more than a few hours in patients implies the placement of a catheter. The length of a clinical block will depend on practical issues such as surveillance, costs, risks of catheter infection [50] or ambulatory surgery. In experimental research, slow release devices in development can already be used to block nerves over a few days [35]. By combining a slow release system (microspheres loaded with bupivacaine and a small amount of dexamethasone) with an entrapment (embedding the spheres in fibrin glue inside a silicon tube), we could achieve a complete sciatic nerve block for a week with complete recovery thereafter [51].

**(b) Selective Block**

As mentioned above, discharges originate in different fiber types depending on the injury model and timing. In clinical setting pain (nociceptive), specific blockade is achieved by reducing the concentration of the local anesthetics. Lots of research is ongoing to discover blockers whose targets are specifically expressed on nociceptors such as TRPV1 or specific isoforms of sodium channels.

TRPV1 can be blocked by an agonist as capsaicin or resiniferatoxin (RTX) which induces a desensitization of the nerve fiber for a longer period. In our hands, RTX directly applied on the sciatic nerve induced a selective block to heat stimulation for 3 days without affecting the response to mechanical stimulation or impairing motor ability. From the 9 voltage-gated sodium channels currently described (Nav1.1–1.9), Nav1.7, 1.8, and 1.9 are almost selectively expressed on nociceptors. No specific blocker of these channels has yet completed clinical trials successfully but compounds are still being tested [52]. These methods of selectively blocking nociceptors are not used in the perioperative setting, yet but in research they are useful tools to study the influence of selective fibers on the mechanisms of pain.

**(c) Evaluation during the Blockade**

Motor/sensitive block and pain levels can be assessed during peripheral or central nerve blockade whereas long-term outcomes as chronic pain or functional recovery can be checked later. We know many plastic changes occur in the perioperative period. In animals, tissue can be harvested during the blockade to study which mechanism are changed.

**(d) Electrophysiological Recordings**

The most important effect of peripheral or central nerve blockade is impeding discharge to pass from periphery to the brain, thereby inhibiting pain. Fundamental research on pathophysiological mechanisms (ectopic electrical activity-dependant or -independent changes, location, and timing of nerve activity) can give hints to clinicians where to block, when, and for how long. Then, clinical trials based on these mechanisms could be designed. Besides observational studies on discharge characteristics in different models, electrophysiological recordings allow to study the effect of blockade on subsequent discharge patterns.

### 3.2. Limits in Animal Research

Behavioral studies in animals are not an easy task. Subject to interobserver and interindividual variability, they integrate many different aspects of the pain pathways (spinal withdrawal reflexes, spino-bulbospinal reflexes as jumping, simple innate behaviors as guarding or licking, or more complicated learned behaviors) depending on the test used. The limits often put forward in animal models are the following: assessment of evoked-pain behavior and not spontaneous pain, no vocalization in the audible range, or no characterization of symptoms. We have to be aware of the limitations when we transfer our results to a clinical application and stay humble when we want to claim the mechanisms in animals and humans are comparable. We hope for mechanistic research in human to improve and to guide our laboratory hypotheses.

## 4. Clues from the Bench

Detailed effects of blocks on nerve injury models are summarized in Table 1 and the description of the models in Figure 1. We will highlight the general ideas below.

### 4.1. Effects of Timing and Duration of Block on Behavior

**(i) Effect of Timing**

The question of whether a nerve blockade has to be effective before or only after surgery is still unresolved. Few experimental studies compared the exact same treatment given before and after lesion. Fletcher et al. demonstrated a better effect on hyperalgesia when injecting bupivacaine in the paw before than after carrageenan [53]. The difference was even sustained when a second dose of carrageenan was injected a week later [54]. This underlines that priming of the nociceptive or sensitive system can be induced by a first injury without being noticed until it is unraveled by the magnified response to a second insult. In a model of intravesical acrolein injection, the timing of lidocaine injection influenced the referred mechanical hyperalgesia on the hindpaws but not the biochemical changes in the bladder itself [55], which points to activity dependant phenomenon or not. Pain mechanisms also change during development: a nerve blockade was done before or after a paw incision in 2-week or 4-week-old rat pups, the preinjury block was only more effective in the 2 weeks old pups [56]. Short block before injury reduced long-term pain-related behavior in the CCI model [57–59], but not in the partial sciatic nerve ligation (PSNL) [57], SNL [60], or spinal nerve cryoneurolysis models [61]. Sometimes the block only delayed heat hyperalgesia in the CCI [62] or mechanical allodynia in the SNL [63]. In the pain clinic, as opposed to the perioperative setting, preventive block cannot be done and we want to know if nerve blockade is useful once pain is established. This is a fundamental pathophysiological issue to know if peripheral inputs still contribute to maintenance of pain or if pain has become a self-maintained central process. Local anesthetic on the dorsal root or spinal nerve after establishment of neuropathic pain could alleviate transiently mechanical and cold allodynia after SNL [34, 60] and inhibiting distal afferent in the CCI model was effective on heat hyperalgesia [64].

The clinical implication of these results is that it is probably useful to perform a nerve blockade before the surgery rather than only starting after. Even if the pain is already established it is worth to use peripheral nerve blockade to test if peripheral nerve activity still participates to the pain process.

**(ii) Effect of Duration**

Apart from the question of when to commence the block, the duration of any perioperative nerve blockade is often questioned. To answer, studies compare the same treatment for short versus long period. In animal inflammatory pain models, repeated injections or bupivacaine-microspheres but not a single injection of bupivacaine reduced pain behavior [65–67]. In the paw incision model, which simulates inflammatory postoperative pain, longer block is more effective for relieving primary and secondary hyperalgesia [68]. For neuropathic pain, a one-week-long peripheral nerve blockade in the SNI model did not prevent pain-related behavior [51], whereas slow release bupivacaine placed at the time of lesion could prevent it in the same model and in the CCI [35].

Clinically, the suspicion that a longer block of nociceptive input could possibly prevent the development of chronic pain states has newly been discussed in the context of phantom limb pain after amputation. While older studies never managed to demonstrate a beneficial effect of epidural or peripheral nerve blocks, a recent observational study revealed astonishingly few patients suffering from phantom limb pain one year after lower limb amputation with prolonged peripheral nerve block performed as peri- and postoperative pain treatment (median duration of block 30 days) [69]. There are clinical and experimental arguments in favor of a long-term block but the duration with the best ratio of risk/benefit has yet to be found.

### 4.2. Biochemical Changes Affected by Blockade

During the period of a regional blockade, behavioral analysis is difficult due to the sensory impairment. Animal research allows observation of changes occurring along the pain pathways during that period by means of tissue collection and analysis. Early signs of neuronal activation in the spinal cord assessed by increased labeling of c-fos (a transcription factor that leads to expression of proteins) is reduced by nerve blockade in inflammatory [70] and postoperative [71] models of pain. Cyclo-oxygenase 2 (COX2) induction and production of prostaglandin E2 (PGE2) in the CNS is also dependent on peripheral nerve inputs [72, 73]. In the SNI model, we found a sciatic nerve blockade which reduces the apoptotic cell death in the spinal cord dorsal horn. This cell death affects inhibitory interneurons. It participates in the disinhibition process involved in the hyperexcitability of the system leading to pain-related behavior. Sadly, the cell death reduction is not long-lasting but only postponed until the end of the block [74]. Microglia and astrocytes, 2 types of glial cells (nonneuronal cell population of the CNS) have been implicated in pain processing [75, 76]. They are generally said to be activated in the context of pain. This activation was reduced in neuropathic pain model through peripheral nerve blockade [77, 78].

The idea of a magic bullet curing all pain has vanished. Therefore, categorizing specific aspects of sensitization processes into activity-dependent or -independent phenomena is useful to know when to use a blockade. These results also favor the concept of multimodal analgesia combining peripheral or central nerve blockade to systemic drugs on various targets.

### 4.3. Effects of Specific Block

In clinical postoperative setting, we adjust the concentration of local anesthetic to obtain a selective nociceptive blockade, which does not block nerve activity arising in thicker myelinated fibers. The paralysis of the limb induced by A-*β* fibers blockade cannot be accepted for a long period due to risk of sore lesions, loss of muscle mass hindering rehabilitation, and masking of complications. Indeed, complications of nerve blockade (nerve injury, hematoma, infection) are suspected when a motor deficit appears or persists [79]. 

In the SNI model, we compared the effect of complete block (using bupivacaine) to specific nociceptive block with RTX (TRPV1 agonist, inducing desensitization of the receptor). Microglial activation was reduced only by the complete block [80]. Tetrodotoxin (TTX) is a sodium channel blocker. Nociceptive fibers contain TTX-resistant sodium channels, and, therefore, myelinated fibers are preferentially blocked by TTX. TTX could prevent neuropathic pain-related behavior after CCI, SNL, and stimulation-induced pain [35, 81], but failed to reduced flinching in an inflammatory model compared to lidocaine [82]. These examples from animal blockade of specific nerve type highlight the paramount importance of thick myelinated fibers in sensitization processes especially in neuropathic pain. Indeed, we mentioned ectopic activity in myelinated axons after injury coincides with tactile allodynia [37]. 

We believe partial blockade such as currently performed, especially with an epidural, could be a reason of failure to prevent chronic postoperative pain despite using pre-emptive long-term block. Older clinical studies already pointed the differential effect of epidural versus spinal intensity of blockade. When both techniques are used at levels were cold and pinprick sensation is abolished, temporal summation is conserved in the epidural group, showing sensitization process might occur in the background of a painless patient [83, 84].

### 4.4. Other Effects of Nerve Block

Local anesthetics have many systemic or local properties besides impeding nerve conduction through voltage-gated sodium channels inhibition.

**(i) The Anti-Inflammatory Effects of Nerve Blockade**

The systemic inflammation tested by the levels of cytokines in the blood is reduced by bupivacaine, and this effect is systemic as ipsi, contralateral block and even contralateral intramuscular injection is effective [85]. In a human model of secondary hyperalgesia, local anesthetic had a systemic effect [86] which is clinically relevant as area of hyperalgesia in the acute postoperative period correlates with the incidence of postoperative chronic pain [87–89].

**(ii) Axonal Transport**

Besides electric discharges axonal transport is another way of signaling a peripheral nerve injury to the CNS. Experimental axonal transport block could influence behavioral and glial changes after nerve injury [90]. Recently bupivacaine has been shown in vivo to inhibit the retrograde transport of TNF*α* after an inflammatory insult [91].

These less known pharmacologic properties of local anesthetics may contribute to the often observed “therapeutic effect” of local anesthetics injection in interventional pain management of certain chronic pain patients, such as facet joint nerve blocks (a) or epidural infiltrations (b), were local anesthetic administration alone often show the same favorable results as their coadministration with corticosteroids. It is also in this context, that previously performed randomized “placebo” controlled trials with negative results comparing the beneficial effect of the combined administration of corticosteroids and local anesthetics with patients receiving local anesthetics alone, as for example for cervical periradicular injections (c), lacked a real placebo group.

## 5. Conclusions and Back to Bedside

In clinical practice, nerve blocks are effective for treatment of acute postoperative pain but their impact on the prevention of chronic postoperative pain shows conflicting results. This paper intended to highlight some of the factors found in experimental studies. The main reason is the blockade limited to nociceptors with absence of blockade of myelinated thicker afferents. The latter account for most of the afferent activity after injury and experimental evidence show they participate in pain related behavior. Perioperative block limited to nociceptive fibers reduces the acute pain and we are maybe missing the sensitization phenomena that occur insidiously at the same time through the myelinated afferents, driving the chronification of the pain process. We mentioned the recent study with long-term block of all fibers [69], although only observational, giving encouraging results on chronic outcomes. For postoperative epidural analgesia, it will not be possible to fully block the inputs over a few days but for peripheral nerve blockade a more intense block can be considered. We have to define the best duration of both peripheral and epidural blockade balancing the advantages of inhibiting some central processes with the risks inherent to these techniques (infection, local anesthetic toxicity, etc.). With regards to the failure of some regional anesthesia techniques we have to keep in mind that some changes might be activity-independent and must, therefore, be addressed by other means. This involves multimodal analgesia combining complementary treatment associating systemic drugs to the regional technique.

## Figures and Tables

**Table 1:** Effect of block on animal nerve injury models. Single means one application, local means on the injury site, and pre-emptive: yes: before the injury. SNL: spinal nerve ligation, CCI: chronic constriction injury, SNI: spared nerve injury, Seltzer: partial sciatic nerve ligation, d: day(s), dpi: day(s) postinjury, iv: intravenous, it: intrathecal, ip: intraperitoneal, ttt: treatment, DRG: dorsal root ganglion, SC: spinal cord, RTX: resiniferatoxin, and TTX: tetrodotoxin.

Author	Year	Drug	Duration	Route	Preemptive	Model	Time of effect	Effect
*Behavioral changes*								

I. Kissin	1999	N-b-tetracaine	single	saphenous	yes	saphenous transection	7 dpi	Prevention early pressure hyperalgesia, caused hyperalgesia alone at 10 d
		Lidocaine	Single	saphenous	yes	saphenous transection	1 dpi	Prevention early pressure hyperalgesia

Y. W. Yoon	1996	Bupivacaine	Single	dorsal root L4/5 5 dpi	no	SNL L5/6	5 dpi	L5: reduction of mechanical + cold allodynia and ongoing pain; L4: reduction of mechanical + cold allodynia

Z. Seltzer	1991	Marcaine	Single	sciatic/ saphenous	yes	sciatic/ saphenous transection		Autotomy is delayed and its magnitude decreased

P. M. Dougherty	1991	Lidocaine	Single	sciatic	yes	CCI	3 and 10 dpi	Reduction in duration and magnitude of thermal hyperalgesia
		Lidocaine	Single	sciatic	yes	Seltzer	3 and 10 dpi	No effect

S. Abdi	2000	Lido/bupivacaine	Single	local before or 4 dpi	yes/no	SNL L5/L6	1 d after ttt	Reduction of mechanical allodynia, no long-term effect

J. M. Zhang	2000	Lidocaine	During 1 or 8 d	DRG following injury	no	DRG compression	1–28 dpi	Reduction of mechanical allodynia and hyperalgesia ipsilaterally with partial effect contralaterally

L. Luo	1995	Lido/tocainide	Single	it	yes	sciatic section	42 d after ttt	No effect on autotomy

S. R. Chaplan	1995	Lidocaine	Single	iv, it, local, 28 dpi	no	L5/L6 ligation	21 d after ttt	Reduction of mechanical allodynia only if plasma concentration was high enough, no long-term effect of local and it

J. Mao	1992	Bupivacaine	Single	sciatic, 3 dpi	no	CCI	1 d after ttt	Reduction of thermal hyperalgesia

J. M. Gonzalez-Darder	1985	Mepivacaine	Single	local	yes	sciatic section	7–70 dpi	Reduction and delay of autotomy

M. L. Sotgiu	1995	Lidocaine	Single	sciatic, iv or iv	yes	CCI	21 dpi	Reduction in paw licking during 2-3 weeks, then no difference

I. Bileviciute-Ljungar	1999	Lidocaine	Repeat	sciatic contra, 6 + 11 dpi	no	CCI	36 dpi	Reduction of thermal hyperalgesia 3-4 d, small effect on pressure stimulation, reduction of autotomy 36 d

T. Yamamoto	1993	Bupivacaine	Single	sciatic	yes	CCI	till 14 dpi	Delaying of thermal hyperalgesia until day 14
		Bupivacaine	Single	sciatic 15 min post	no	CCI	7 dpi	No effect on thermal hyperalgesia

M. R. Suter	2003	Bupvacaine	Long term	sciatic/spheres	yes	SNI	4 weeks	No effect on mechanical allodynia, thermal hyperalgesia, cold allodynia

Y. S. Lyu	2000	TTX	Single	DRG	no	Chung L5 ligation	2 h after ttt	Reduction of mechanical allodynia, no long-term effect

S. R. Chaplan	2003	ZD7288	Single	ip, 7 dpi	no	SNL L5/6	1 day after ttt	Reduction in mechanical allodynia for 2 h, no effect at 24 h

L. M. Batista	2009	Lidocaine	Single	sciatic	yes	CCI, nylon	over 28 days	Reduction of scratching, thermal hyperalgesia (noxious and non-noxious)

I. Sukhotinsky	2004	Lidocaine	Single	DRG L4 or L5	no	SNL	280 min	Reduction allodynia from 2 to 280 min after ttt, more effective on L5 than on intact L4

S. Eschenfelder	2000	Lidocaine	Single	dorsal root L5 before section	yes	SNL L5	57 dpi	No difference for mechanical hyperalgesia

*Biochemical or electrophysiological changes*								

J. M. Zhang	2004	Lidocaine	7 d	ip, pump	no	SNL	7 and 14 dpi	Reduction in tyrosine hydroxylase staining
		Lidocaine	14 d	sciatic, pump	yes	sciatic transection	14 dpi	Reduction in tyrosine hydroxylase staining

C. T. Lin	2009	Lidocaine	Single	median nerve	yes	median nerve transection	28 dpi	Dose dependent reduction of injury discharge pre and post electrical stimulation and of NPY and c-fos in cuneate nucleus

I. Omana-Zapata	1997	TTX	Single	intravenoous	no	sciatic transection	4–10 days	Dose dependent reduction of ectopic activity

I. Bileviciute-Ljungar	2001	Lidocaine	Single	contralateral subcutaneous	no	CCI	14 dpi	WDR L4/5 neuron ipsilateral: spontaneous hyperactivity reduced for 60 min

L. A. Colvin	2001	Amethocain	Single	dorsal roots L2–6	no	CCI	10–14 dpi	No effect on neuropeptide Y release in spinal cord (measurement period of 2 h)

J. Scholz	2005	Bupivacaine	7 d	sciatic, spheres	yes	SNI	7 dpi	Delay in apoptosis of inhibitory interneurons in the dorsal horn of spinal cord

Y. R. Wen	2007	Bupvacaine	3 d	sciatic, spheres	yes	SNI	3 dpi	Inhibition of p38MAPK activation in microglia in the spinal cord dorsal horn

W. Xie	2009	Bupivacaine/TTX	Long term	sciatic/DRG pump 7d	no	SNI/SNL	1–10 dpi	TTX: inhibition of NGF increase (DRG, d3) OX-42 (SC, d3) and GFAP (SC, d10); both: inhibition of glial activation (DRG, d1–10)

S. I. Chi	1993	Local anesthetic		sciatic or systemic	no	sciatic transection	2 and 14 dpi	Reduction in c-fos immunoreactivity in dorsal horn of spinal cord

S. I. Chi	1993	Lidocaine		sciatic or systemic	yes	sciatic transection	2 dpi	Reduction in c-fos immunoreactivity in dorsal horn of spinal cord

M. R. Suter	2009	Bupivacaine/RTX	2 d	sciatic, spheres	yes	SNI	2 dpi	Bupi: inhibition of microglia proliferation and p38MAPK activation in dorsal horn of spinal cord; RTX: no effect

*Mixed outcomes*								

L. Liang	2010	TTX	Repeat	sciatic, daily	yes	electrical stimulation	up to 35 dpi	Reduction of mechanical allodynia, GFAP-staining on DRG

B. A. Rooney	2007	Lidocaine	Single	dorsal root	yes	bilateral dorsal root L4/5 section	up to 13 days	No increase in excitatory amino acid 10 min post injury, reduction in mechanical allodynia

W. Xie	2005	Bupivacaine	Long term	sciatic, after lesion	no	CCI and SNI	up to 70 d (CCI), 150 d (SNI)	Reduction in mechanical and heat pain for 60 d (CCI + SNI), suppression of hyperactivity at 20–28 dpi in A and C fibers

W. Xie	2005	TTX	Long term	sciatic, TTX (pump 3 or 7 d) just after lesion or 10 d later	no	CCI and SNI	up to 70 d (CCI), 150 d (SNI)	Reduction in mechanical and heat pain for 60 d (CCI + SNI), TTX 10 d effective only during infusion, suppression of hyperactivity at 20–28 dpi in A and C fibers

R. W. Colburn	1997	Bupivacaine	Repeat	spinal nerve before cut + before closure	yes	spinal nerve cryoneurolysis	10 dpi	Reduction of microglial, but only minimal on astrocytic response, no effect on mechanical allodynia

S. Lee	2007	Lidocaine	Single	spinal nerve, it	yes	SNL L5/6	1–4 dpi	Delay in mechanical allodynia by 1–4 d

C. Sato	2008	Ropivacaine	Repeat	epidural, daily 7–17 dpi	no	CCI	since 11 dpi	Relief of thermal hyperalgesia, small reduction of mechanical allodynia, NGF increase in DRG with ropivacaine

W. Xie	2007	TTX	7 d	sciatic, pump	yes	sciatic transection	35–49 dpi	Reduction of hyperexcitability of large and medium cells and sympathetic sprouting. No change in C fiber through TTX
